# Concurrent nephrotic syndrome and acute renal failure caused by chronic lymphocytic leukemia (CLL): a case report and literature review

**DOI:** 10.1186/1746-1596-6-99

**Published:** 2011-10-13

**Authors:** Xianrui Dou, Haitang Hu, Yongle Ju, Yongdong Liu, Kaifu Kang, Shufeng Zhou, Wenfang Chen

**Affiliations:** 1 Department of Nephrology, the Affiliated Shunde First People's Hospital of Southern Medical University, Penglai Road, Daliang District, Foshan 528300, China; 2 Department of Pathology, the First Affiliated Hospital of Sun Yat-sen University, 58# Zhongshan Road II, Guangzhou 510080, China; 3 Department of Pharmaceutical Sciences, College of Pharmacy, University of South Florida, Tampa, Florida 33612, USA

## Abstract

Kidney injury associated with lymphocytic leukemia (CLL) is typically caused by direct tumor infiltration which occasionally results in acute renal failure. Glomerular involvement presenting as proteinuria or even nephrotic syndrome is exceptionally rare. Here we report a case of 54-year-old male CLL patient with nephrotic syndrome and renal failure. The lymph node biopsy confirmed that the patients had CLL with remarkable immunoglobulin light chain amyloid deposition. The renal biopsy demonstrated the concurrence of AL amyloidosis and neoplastic infiltration. Combined treatment of fludarabine, cyclophosphamide and rituximab resulted in remission of CLL, as well as the renal disfunction and nephrotic syndrome, without recurrence during a 12-month follow-up. To our knowledge, this is the first case of CLL patient showing the nephrotic syndrome and acute renal failure caused by AL amyloidosis and neoplastic infiltration. Though AL amyloidosis caused by plasma cell dyscrasia usually responses poorly to chemotherapy, this patient exhibited a satisfactory clinical outcome due to successful inhibition of the production of amylodogenic light chains by combined chemotherapy.

## Background

AL amyloidosis is characterized by widespread, progressive deposition of amyloid fibrils derived from monoclonal immunoglobulin (Ig) light chains, leading to multi-system organ failure among which the kidneys and heart are most frequently affected [1]. Although AL amyloidosis is typically caused by plasma cell proliferative disease especially multiple myeloma, it can also be caused by other lymphoproliferative disorders of B-cells such as lymphoplasmacytic lymphoma, of which the neoplastic B cells produce monoclonal immunoglobulin light chains [2]. Only few cases of AL amyloidosis associated with chronic lymphocytic leukemia (CLL) have been reported [2-5]. Another relatively common injury caused CLL is the direct neoplastic cell infiltration as demonstrated by autopsy studies [6], but acute renal failure due to severe infiltration is rare [7-9].

In this report, we present a patient with nephrotic syndrome and renal failure associated with CLL. The patient completely recovered from the nephrotic syndrome and renal dysfunction after CLL was controlled with chemotherapy. We have reviewed the literature and discussed the relationship between CLL and amyloidosis and the pathological implications.

## Case presentation

### Clinic data

A 54-year-old male Chinese patient was admitted to the Shunde People's hospital on March 7, 2008, complaining of nocturia and edema of face and both lower extremities for more than two months. The patient had no history of any renal disease. Physical examinations revealed normal vital signs, moderate hypertension (162/96 mmHg) and pitting edema of the lower extremities. Enlargement of lymph nodes involving inguinal, axillary, submaxillary and supraclavicular fossa with a diameter of more than 1 cm was found. The remainder of the examination was unremarkable.

**Figure 1:**
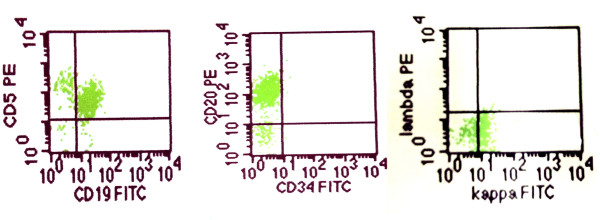
**Flow cytometric analysis of the bone marrow aspiration**. The proliferated lymphocytes were positive for CD5, CD20, CD19 and kappa light chain.

**Figure 2:**
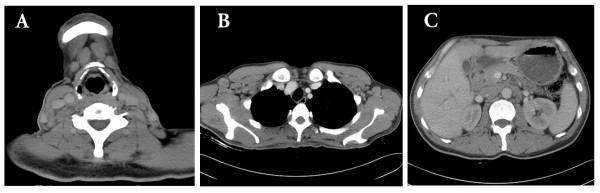
**Computed tomography scanning of the neck, chest and abdomen (A)**. Enlargement of numerous lymph nodes in the neck. (B). Numerous enlarged lymph nodes in axillary space. (C) Enlargement and confluence of lymph nodes in the peritoneal cavity.

Both his hemoglobin and erythrocyte count were in normal range. The leukocytes increased to 16.8 × 10^9^/L with elevated lymphocytic proportion by 61.8% (normal range: 24%-40%). Urine analysis showed proteinuria of 5 g/L with a RBC count of 150/μL. His 24-hour total urinary protein excretion was 5.11 g and serum albumin level was 38 g/L. The blood creatinine (Cr) concentration was 290.04 μmol/L (normal range: 53-115 umol/l) and the urea nitrogen (BUN) was 13.49 mmol/L (normal range: 2.9-8.6 mmol/l) indicating the impaired renal function. Both Ig-M (kappa) monoclonal protein and free kappa were detected in his serum by immunofixation electrophoresis, but urine Bence Jones protein was negative. He also had high serum IgM concentration (3.9 g/L, normal range: 0.50-2.20 g/L) while the IgG and IgA levels were normal. The serum complement 3 decreased to 0.03 g/L (normal range: 0.79-1.17 g/l). The tests for antinuclear antibody, rheumatoid factor, cryoglobulin and hepatitis virus B and C all showed negative. The bone marrow aspiration showed increased cellularity and the accumulation of small immature-appearing lymphocytes, without increase of plasma cells. Flow cytometric analysis showed that those lymphocytes were positive for CD5, CD19, CD20, HLA-DR, kappa and surface membrane IgM, and negative for CD3, CD7, CD10, CD38 and lambda (Figure 1). The CT scanning revealed numerous intumesced lymph nodes in the neck, thoracic cavity, celiac and retroperitoneal space (Figure 2). Liver and spleen were both enlarged with dense and fine sonogram. The echocardiography indicated mildly and symmetrically thickened left ventricular wall. The patient was diagnosed with acute renal failure together with a nephrotic-range proteinuria.

### Pathological findings

**Figure 3:**
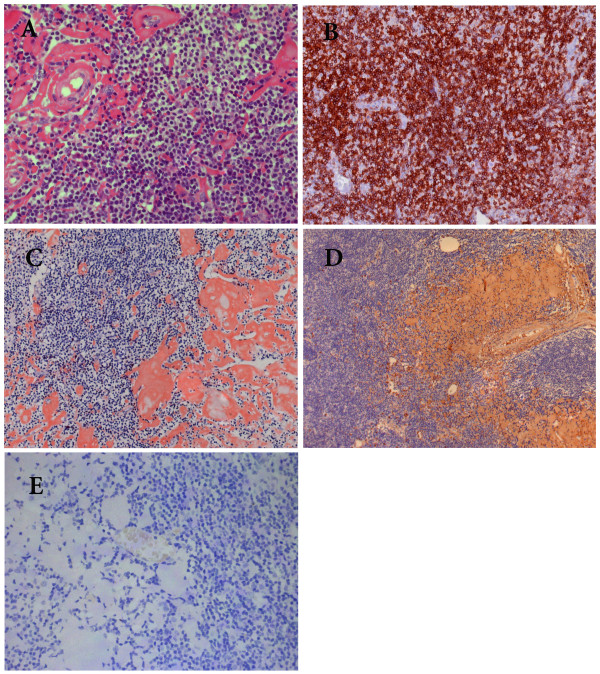
**Photomicrographs of histological changes of the affected lymph node**. (A). Lymph node involved showing diffuse small lymphocyte-like cells and patches of homogenous materials in the dark background of proliferating cells. (HE staining×400). (B) The proliferating cells are CD5 positive by immunohistochemistry (×200). (C). The deposit was strongly Congon-Red positive. (×200). (D). Strong positivity of κ light chain demonstrated by immunohistochemistry (×100). (E). The deposit was completely negative for λlight chain. (×200).

The biopsy from one of enlarged lymph nodes showed effacement of the architecture with a dark background of cells consisting of numerous small lymphocyte-like cells with scattered larger cells including prolymphocytes and paraimmunoblasts. The predominant cells were slightly larger than normal lymphocytes (Figure 3A) with round nucleolus and clumped chromatin. Only mild nuclear irregularity was noted. The mitotic phase was occasionally observed. Immunohistochemistry showed these cells were positive for CD20, CD79α, CD5 (Figure 3B), and CD23 but negative for CD3, CD45RO, CyclinD1, CD38, and CD138. An important finding was that there were patches of homogeneous hyaline material in the interstitium and vascular walls (Figure 3A), which was verified to be amyloid protein by Congon-red staining (Figure 3C). Immunoassay confirmed that the deposit was positive for kappa but negative for lambda and serum amyloid A protein (Figure 3D-E). The patient was diagnosed as CLL with systemic AL amyloidosis.

**Figure 4:**
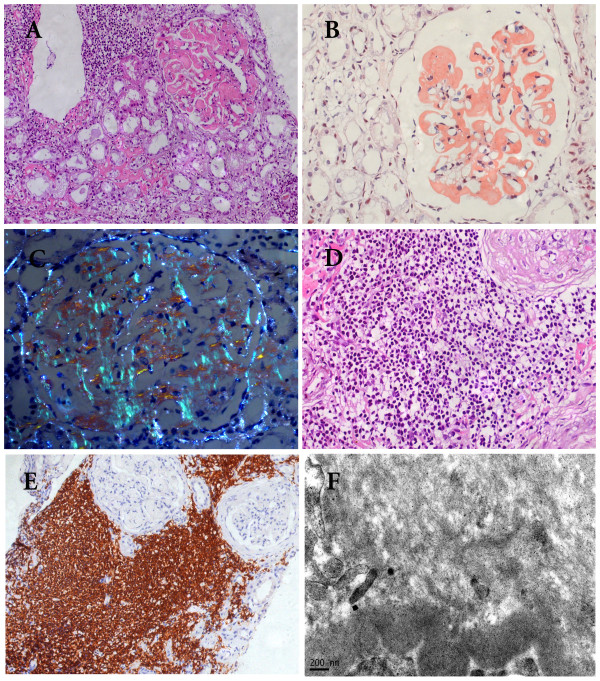
**Photomicrographs of histological changes of the renal biopsy**. (A) Renal biopsy illustrated small lymphocyte-like cell infiltrate in the interstitial tissue. The glomeruli showed amorphous eosinophilic material in the meangium and extending to the peripheral capillary walls leading the obliteration of segmental capillary loop. Foci of deposition were found in the interstitial and peritubular space. (HE staining×200). (B). Salmon pink staining of the glomeruli with Congo red staining.(×400). (C). Apple green birefringence was detected under polarized light (×400). (D) Uniform small lymphoid cell infiltrate in a nodular pattern around a sclerotic glomerulus. (HE staining×200). (E) The infiltrating cells in the interstitium were CD20 positive by immunohistochemistry (×200). (F) Non-branched and randomly arranged fine fibrils in a diameter of 10 nm were found in the glomerular mesangial area (×37000).

Because of unknown reason of worsening of proteinuria and decreasing of the Alb down to 29 g/l after 2 courses of combinative chemotherapy including 2 mg chlorambucil and 30 mg prednisone, a kidney biopsy was performed. The patient's renal tissue submitted for light microscopy consisted of two cores of renal cortex and medulla containing 35 glomeruli. There was no global or segmental sclerosis. All glomeruli showed diffused, eosinophilic and homogeneous material in mesangial area replacing the normal mesangial matrix (Figure 4A). Some extended into the peripheral capillary walls and appeared as large subendothelial deposits or even seemed to be located in the capillary lumen. The deposits were amyloid as shown by strong Congo-red positive staining (Figure 4B) and bright apple green birefringence under polarized light (Figure 4C). The same deposits were found in the interstitium, peritubular space and blood vessel walls. Immunohistochemistry showed that the amyloid was kappa positive but negative for lambda and serum amyloid A protein. Only mild focal tubular atrophy was found. Clusters of lymphocyte-like cells which were CD5 and CD20 positive infiltrated in renal interstitium, occupying less than 30% of the area of the cortical tissue examined (Figure 4D and 4E). The small arteries exhibited mild fibrointimal thickening and the arterioles appeared segmental hyalinosis. Immunofluorescence staining showed faint staining of IgG and IgM along the peripheral capillary in glomeruli while IgA, C3, C1q and fibrinogen were negative. Non-branched fibrils with 10 nm-diameters were detected in mesangium and subendothelial area (Figure 4F) by electronic microscopy. All these histological changes indicated a direct infiltration of the tumor cells in the renal parenchyma together with AL amyloidosis.

### Treatment and following-up

Chemotherapy was given by combination of 44 mg fludarabine and 20 mg dexamethasone from July 24, 2008 to September 26, 2008 for 3 courses of treatment. After that, the lymph nodes size decreased and the edema was dramatically ameliorated with decreased serum Cr (200 μmol/L), increased serum albumin (39.96 g/L), declined serum IgM level (2.38 g/L) and slightly reduced 24-hour urinary protein excretion (4.22 g/24 h). However, the patient developed a severe bone marrow depression defined by markedly reduced blood cell count (erythrocytes: 1.66 × 10^12^/L, leukocytes: 0.62 × 10^9^/L, platelet: 21 × 10^9^/L). Then the chemotherapy regimen was adjusted (fludarabine 50 mg, CTX 0.3 g and rituximab 600 mg). After 3 courses of treatment from Nov 27, 2008 to Feb 19, 2009, the patient achieved a satisfactory hematologic response. His serum IgM concentration gradually decreased to the normal level and the monoclonal immunoglobulin was undetectable by serum immunofixation electrophoresis. At the same time, the patient experienced a gradual improvement in clinical status and decrease in the proteinuria and serum creatine. His renal function, urinary protein and serum C3 level were recovered entirely in Sept 2009 (24-hour urinary protein excretion: 139.20 mg/24 h, urine routine test: negative, serum Cr: 121.00 μmol/L, serum C3 level 0.96 g/L).

## Discussion

Proteinuria caused by direct infiltration of neoplastic cells is generally mild. Severe proteinuria or even nephrotic syndrome often suggests glomerular involvement [7,10]. Though CLL is rarely complicated with nephrotic syndrome, a spectrum of glomerular diseases including light chain deposition disease (LCDD), AL, membranoproliferative glomerulonephritis (MPGN) [11], membrane nephropathy (MN) and minimal change disease (MCD) have been reported, which suggests the presence of various pathogenesis pathways of CLL-associated nephrotic syndrome[2,7,11-13]. The glomerular injuries may be directly caused by the lymphoplasmacytic neoplasm through a paraprotein deposition process which finally results in amyloidosis or monoclonal immunoglobulin deposition disease or the indirect immune-mediated mechanisms may also be involved. In our case, the monoclonal Ig light chain detected in the sera was the same class as that found on the surface membrane of lymphocytes and the amyloid in the kidney and lymph node, indicating that the monoclonal Ig originated from the proliferating cells. Based on this finding as well as the histopathological observations, we made a diagnosis of CLL associated AL amyloidosis. The reason that we could not detect monoclonal Ig light chain in the lymphocytic cells by immunohistochemistry may be ascribed to the inaccessibility of epitope after tissue fixation.

Histologically, the leukaemic infiltratation in the kidney can be nodular or interstitial in CLL. In this patient, both patterns were observed. When the infiltration is nodular, especially when there are clusters of cells around sclerotic glomeruli or atrophic tubules, it is important to determine whether it is tumor infiltration leading to their destruction or is only local inflammation secondary to glomerular scelerosis and tubular atrophy caused by other glomerulopathies. The mixture of various inflammatory cells generally indicates the secondary local inflammatory response, while the monotonous appearance of the cells usually suggests tumor infiltration. But when it comes to a biopsy specimen, it is difficult to distinguish both situations due to the local distribution of the infiltration. In this regard, further immunohistochemical examinations should be applied. In general, leukemic infiltration in the kidney should be considered when a patient with CLL presents with renal impairment. This kind of patients usually responds well to chemotherapy as seen in our case [8,14,15].

AL amyloidosis can be complicated with any clonal B cell dyscrasia, especially plasma cell dyscrasia. The misfolded monoclonal free light chains deposited in the extracellular space in a fibrillar form which are very hard to be absorbed. Accumulation of these fibrils in the kidney causes progressive renal impairment with proteinuria, even renal failure. It is well known that most amyloid depositions associated with plasma disorders are lambda-light chains [16]. On the contrary, in AL amyloid caused by CLL, the monoclonal light chains detected are predominantly kappa type as in our case [3,5]. Though AL amyloidosis caused by plasma cell dyscrasia often has a poor prognosis with a median survival of 35.2 months from diagnosis due to the poor response to chemotherapy [17], CLL-associated AL amyloidosis were reported to be significantly alleviated and obtained a prolonged survival of more than 7 years [2]. As with our patient, his proteinuria decreased very slowly and did not resolve until one year after he obtained hematologic remission. This is believed to be associated with the reduced production of amyloidogenic light chains so that the clearance of existing deposits exceeds the rate of deposition. Those deposited amyloids were gradually mobilized and cleared by the body.

Another interesting finding is that the serum concentration of C3 in our patient changed significantly after chemotherapy. It was in the lowest level when the patient was in acute phase and the serum IgM was high. With the decrease of serum IgM after treatment, it recovered to the normal level, indicating that impaired complement system might be involved in the pathogenesis of CLL and its complications [18].

## Conclusion

We herein first reported a rare case of nephrotic syndrome combined with acute renal failure caused by CLL. From our patient and few cases reported in the literature, we suggest that AL amyloidosis should be considered in CLL patients presenting with proteinuria or nephrotic syndrome. AL amyloidosis associated with CLL may have a more favorable prognosis than those caused by multiple myeloma due to a better response to chemotherapy. Further investigations are needed to better clarify the clinical outcomes and prognosis of this unusual complication of CLL and to better understand the pathological and molecular mechanisms of CLL- induced nephrotic syndrome.

## Consent

A written informed consent was obtained from the patient for publication of this case report and any accompanying images. A copy of the written consent is available for review by the Editor-in-Chief of this journal.

## Competing interests

The authors declare that they have no competing interests.

## Authors' contributions

XRD has drafted the manuscript. HTH and YLJ have made contributions to acquisition of clinical data. YDL and KFK have carried out the tissue staining, immunoassays and electronic microscopic examination. SFZ has revised the manuscript carefully. WFC has made analysis of the histological features and the clinical-pathological relations. All authors read and approved the final manuscript.
